# Supragingival Microbiota Alterations in Individuals With Sleep Bruxism: A Pilot Study

**DOI:** 10.1111/joor.13983

**Published:** 2025-04-29

**Authors:** Athénaïs Collard, Alban Mathieu, Paolo Landa, Annik Pelletier, Gilles J. Lavigne, Arnaud Droit, Nelly Huynh, Vanessa P. Houde

**Affiliations:** ^1^ Faculté de Médecine Dentaire, Groupe de Recherche en Ecologie Buccale (GREB) Université Laval Quebec City Canada; ^2^ Centre de Recherche du CHU de Québec‐Université Laval Site CHUL Quebec City Canada; ^3^ Département d'Opérations et Systèmes de Décision Université Laval Quebec City Canada; ^4^ Faculté de Médecine Dentaire Université Laval Quebec City Canada; ^5^ Faculté de Médecine Dentaire Université de Montréal Montreal Canada; ^6^ Faculty of Dental Medicine and Oral Health Sciences and Faculty of Medicine, Neurology & Neurosurgery McGill University Montreal Canada

**Keywords:** 16S rRNA gene sequencing, computational biology, sleep bruxism, supragingival microbiota

## Abstract

**Background:**

Sleep bruxism (SB) is an oral behaviour associated to jaw clenching or grinding of the teeth. Its aetiology is most likely multifactorial; however, recent studies suggested that SB is associated with activation of the sympathetic nervous system. Dysbiosis of the oral microbiota is linked to oral and systemic diseases. The relationship between supragingival microbiota and SB remains unexplored.

**Objective:**

This study aimed to investigate the association between SB and the composition of the supragingival microbiota.

**Methods:**

Nineteen metabolically and orally healthy subjects were recruited. After SB diagnosis, supragingival microbiota samples were collected. Microbial DNA was extracted and subjected to 16S rRNA gene sequencing. Amplicon sequence variant (ASV) analysis method was used to correlate the composition of supragingival microbiota with SB.

**Results:**

Bacterial diversity decreased in the SB group. ASV_200 (*Actinomyces*) and ASV_94 (*Morococcus*) were enriched in the SB individuals, whereas ASV_405 (*Morococcus*) was enriched in the controls. The role of the decreased bacterial diversity as well as the enrichment of specific ASV in the mechanism explaining the genesis of SB remain to be determined.

**Conclusion:**

The differences in the composition of the supragingival microbiota may lead to the assessment of important questions in the fields of oral microbiota composition and sleep medicine.

## Graphical abstract

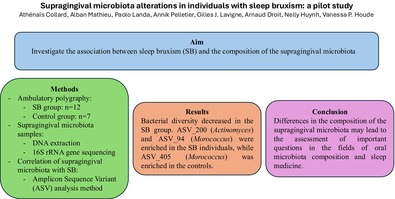

## 1 Introduction

Sleep bruxism (SB) is a complex oral behaviour that can be defined by ‘repetitive masticatory muscle activity characterized by clenching or grinding of the teeth and/or strengthening or thrusting of the mandible’ [1]. Rhythmic masticatory muscle activity (RMMA) occurs in 60% of the population during sleep [2]. However, RMMA is three times more frequent and exhibits greater muscle contraction amplitude in bruxers compared to non‐bruxers [3, 4]. SB can lead to excessive wear of tooth enamel, cracks, fractures and tooth mobility [5]. It is also associated with muscular tension in the jaw and neck region as well as with pain/fatigue of the masticatory muscles (masseter, temporalis and pterygoid muscles) and headache [6, 7]. Although the aetiology of SB remains under investigation, there is growing evidence suggesting its association with activation of the autonomic nervous system, particularly the sympathetic nervous system [8, 9]. Another possible aetiology factor of SB is genetic predispositions [10]. However, more studies are required to confirm the role of genetic in the aetiology of SB.

The oral microbiota refers to the complex ecological community of commensal, symbiotic and pathogenic bacteria present in the human oral cavity. This dynamic ecosystem is crucial for oral health and overall body health [11]. Microorganisms in the oral cavity are not randomly distributed but are functionally and spatially organised in oral habitats (or oral ecological niches), which provides the nutrients and optimal microenvironment for its specific population [12]. Dental hygiene, diet, age, genetic, pregnancy, ethnic origin and systemic health (such as type 2 diabetes) are parameters that can influence the composition of the oral microbiota [13, 14, 15, 16, 17]. Amongst the oral ecological niches, the supragingival microbiota (or supragingival dental plaque) is located on tooth surfaces above the gum line and is composed of bacteria organised in biofilm [18]. Healthy supragingival microbiota is predominantly composed of bacteria from the genera *Streptococcus*, *Actinomyces*, *Veillonella* and *Corynebacterium* [19]. Dysbiosis of the supragingival microbiota can lead to conditions such as dental caries [20].

The link between the oral microbiota and the brain/nervous system (often referred to as the mouth–brain axis) is an emerging field of research in microbiology and neuroscience. Recent research suggested that the oral microbiota may have implications beyond oral health, potentially influencing the brain and the central nervous system [21]. Dysbiosis of the oral microbiota has been associated with Alzheimer's disease [22] and to the progression of dementia [23]. In a paediatric population, the abundance of bacteria within the oral microbiota has been shown to be highly dynamic, fluctuating during sleep [24]. Furthermore, a recent study has compared the composition of salivary microbiota from individuals with SB to controls [25]. However, no study has yet determined whether the oral microbiota and its metabolites can communicate with the brain and influence its functions.

To our knowledge, the relationship between the supragingival microbiota and SB, as well as their potential effects on the brain and the nervous system, is a novel avenue to further decrepit the mechanisms and putative causes of SB. Therefore, this study aimed to investigate the association between SB and supragingival microbiota composition.

## 2 Methods

### 2.1 Subjects

The project was conducted in accordance with the Declaration of Helsinki and received approval from the Research Ethics Committees of Université Laval (ethics number: 2021‐120 R‐3). Participants were recruited through an announcement email from Université Laval. Potential volunteer candidates were initially screened via phone interviews conducted by research staff. Prior to inclusion in the study, all participants provided informed consent. Candidates who meet the inclusion criteria were invited to the university dental clinic for a clinical examination.

Inclusion criteria were age from 18 to 35 years, good metabolic health (no diagnosis of metabolic diseases), a normal body mass index (18.5–24.9) and good oral health (absence of plaque‐induced gum inflammation, periodontal disease, dental caries and endodontic lesions). Participants with depression, anxiety, schizophrenia, Parkinson's disease, epilepsy or sleep‐related illnesses (insomnia, sleep apnea) were excluded. Additionally, individuals who had taken substances or medications affecting sleep (antidepressants, antipsychotics, narcotics, central nervous system stimulants, corticosteroids, etc.) or the microbiota (antibiotics, prebiotics, probiotics) within 3 months prior to the study were also excluded.

### 2.2 Ambulatory Polygraphy

During their visit to the dental clinic, participants filled out questionnaires to assess history of SB, general health and sleep quality. The clinical examination was performed by a calibrated clinician and included dental, temporomandibular joint and masticatory muscle status [8]. Participants were sent home for two consecutive nights recording (level 3) with an ambulatory polygraphy (PG) device to confirm or infirm SB diagnostic. The ambulatory PG system (Medibyte, Braebon Medical Corporation, Ottawa, ON, Canada) included cardiorespiratory channels and unilateral electromyography channels of masseter and temporalis muscles. PG data were scored by the same examiner, blind to participant status [26, 27]. Subjects received the PG instrumental‐based diagnosis of SB when they had > 4 episodes per hour of sleep [28].

### 2.3 Based on the Clinical Examination and PG Results, Participants Were Assigned to One of the Two Experimental Groups

SB group: positive on three criteria, that is, self‐report, clinical examination and ambulatory PG instrumental, OR, at least positive on self‐report or clinical examination findings.

Control group: negative on self‐report SB and clinical signs and PG instrumental assessments.

### 2.4 Statistical Analyses

The number of participants was estimated based on a study with a similar recruitment process [29]. According to this study, we estimated, using the sensitivity and accuracy indices of the diagnostic criteria for SB, that 45 participants would need to be retained at the first stages of selection (questionnaires and clinical examination) to obtain a total of 20 correctly diagnosed participants at the final stage (PG diagnosis of SB).

The statistical analyses were performed using RStudio (version 2023.06.1 Build 524) (Boston, MA, USA). Categorical variables were summarised as numbers and proportions, and associations between variables were determined using the Chi‐square test. The Shapiro–Wilk test was performed on all continuous variables to test the normality of the distribution. Groups comparison was then performed with a Student *T* test. Statistical significance was set at *p* < 0.05.

### 2.5 Supragingival Microbiota Collection

Supragingival microbiota samples were collected after participants abstained from oral hygiene for 12 h, at the end of their visit to the dental clinic. Supragingival dental plaque from the buccal and lingual surfaces of all teeth were collected by a calibrated clinician with sterile curettes (Gracey standard periodontal curette; Hu‐Friedy, Chicago, IL, USA) and pooled to increase bacterial diversity. Each pooled dental plaque sample was then transferred into a sterile tube containing a DNA/RNA shield solution (Zymo Research, Irvine, CA, USA) and stored at −80°C until DNA extraction.

### 2.6 Bioinformatic Analyses

#### 2.6.1 16S rRNA Gene Sequencing

Bacterial DNA from each pooled microbiota (supragingival dental plaque) sample was extracted by using the ZymoBIOMICS extraction kit from Zymo Research (Irvine, CA, USA). The concentration of DNA was determined with a NanoDrop device (ThermoFisher, Mississauga, ON, Canada). The DNA sequencing (16S rRNA gene sequencing) was performed by the Centre d'expertise et de services Génome Québec (Montreal, QC, Canada) on an Illumina MiSeq apparatus using v3‐v4 primers (PE 300 bp [100 000 reads]). The DNA sequencing data were deposited in the Sequence Read Archive (SRA) GenBank database server under accession number: PRJNA1138524.

#### 2.6.2 16S rRNA ASV Analysis Method

A mean of 99 780 16S sequences per sample were analysed with an amplicon sequence variant (ASV) methodology using DADA2 pipeline. Briefly, the 16S amplicons were trimmed for their adapters using cutadapt and the options (‐m 270 ‐M 330 ‐‐discard‐untrimmed). The remaining reads were processed using DADA2 commands [30]. The dada2 pipeline allowed to filter and trim the data based on their quality profile and removed chimera using an error model based on the sequenced data. A count table of the retrieved ASVs was achieved using the makeSequenceTable function. ASVs generation pipeline resulted in the generation of a mean of 40 658 dechimerised and merged reads by sample.

#### 2.6.3 Taxonomic Annotation of ASVs

The ASVs were analysed using Blastn v2.4.0 against RefSeq 16S database and only best hit were kept (with a minimum similarity threshold of 95%). Remaining ASVs were let with no taxonomy association. The analysis and data representation were produced using R4.2.2. ASVs counts were normalised using the total annotation depth with the decostand function of the vegan package (v2.6‐4) to produce relative abundance graphs. ASVs counts were also used using the DESeq2 R package in order to normalise counts and produced a biplot using the prcomp function of the stats package.

#### 2.6.4 ASV Differential Analysis

Univariate analysis has been achieved with the ASV raw count table and the DESeq2 model [31]. The 12 samples from the bruxism group were compared to the seven samples of the control group to look for ASV with a differential distribution between the groups.

## 3 Results

### 3.1 Demographic, Clinical and Dental Characteristics

Twelve participants with SB (7 men and 5 women) and seven control subjects (2 men and 5 women) were recruited for this study (Table 1). All participants completed the study with no dropouts. The mean age and body mass index (BMI) were 28.92 ± 5.69 years and 24.81 ± 3.62 kg/m^2^ for the bruxism group and 24.71 ± 3.64 years and 22.69 ± 3.32 kg/m^2^ for the control group. During the clinical examination, no group differences were found for total tooth wear score, history of tooth grinding or clenching, masseter hypertrophy, temporomandibular joint (TMJ) noise, hyperkeratinized lesion of the jugal mucosa and scalloped tongue (Table 1).

**TABLE 1:** Study groups and clinical parameters for overall mean (SD) or number of demographic, clinical and dental characteristic assessed during the clinical examination.

		B (*n* = 12)	C (*n* = 7)	*p*
Sex (M/W)		7/5	2/5	0.35
Age (years)		28.92 (5.69)	24.71 (3.64)	0.05
BMI (kg/m^2^)		24.81 (3.62)	22.69 (3.32)	0.194
Total tooth wear score		3.41 (2.94)	2.57 (2.69)	0.524
Aware of grinding your teeth	No	10	7	1.0
	Yes	2	0	
Aware of clenching teeth	No	5	5	0.333
	Yes	7	2	
Masseter hypertrophy	No	8	5	0.592
	Unilateral	1	1	
	Bilateral	3	1	
TMJ noise	No	10	6	0.792
	Unilateral	1	0	
	Bilateral	1	1	
Hyperkeratinized lesion of the jugal mucosa	No	7	4	1.0
	Yes	5	3	
Scalloped tongue	No	4	4	0.546
	Yes	8	4	

Abbreviations: B, bruxism group; BMI, body mass index; C, control group; M, man; SD, standard deviation; TMJ, temporomandibular joint; W, woman.

### 3.2 Sleep Variables and Sleep Bruxism

Sleep variables recorded with the ambulatory PG are presented in Table 2. No group differences were observed in total sleep time. We also calculated the number of RMMA/SB episodes/night and the RMMA/SB index. Individuals with SB experienced significantly more bruxism episode per night compared to the control subjects. Additionally, the RMMA/SB index was significantly increased in the SB group, which is in line with a previous study using ambulatory PG for SB diagnosis [28].

**TABLE 2:** Polygraphic variables for overall mean (SD) of sleep variables and SB.

PG variables	B (*n* = 12)	C (*n* = 7)	*p*
Total sleep time (min)	399.7 (82.9)	413.2 (71.5)	0.484
Total number of RMMA/SB episodes/night	52.1 (29.6)	25.1 (8.1)	**0.003**
RMMA/SB index (number/h)	7.5 (2.78)	3.6 (1.1)	**< 0.0001**

*Note:* Significant differences are in bold.

Abbreviations: B, bruxism group; C, control group; PG, polygraphy; RMMA, rhythmic masticatory muscle activity; SB, sleep bruxism; SD, standard deviation.

### 3.3 Supragingival Microbiota Analyses

**FIGURE 1:**
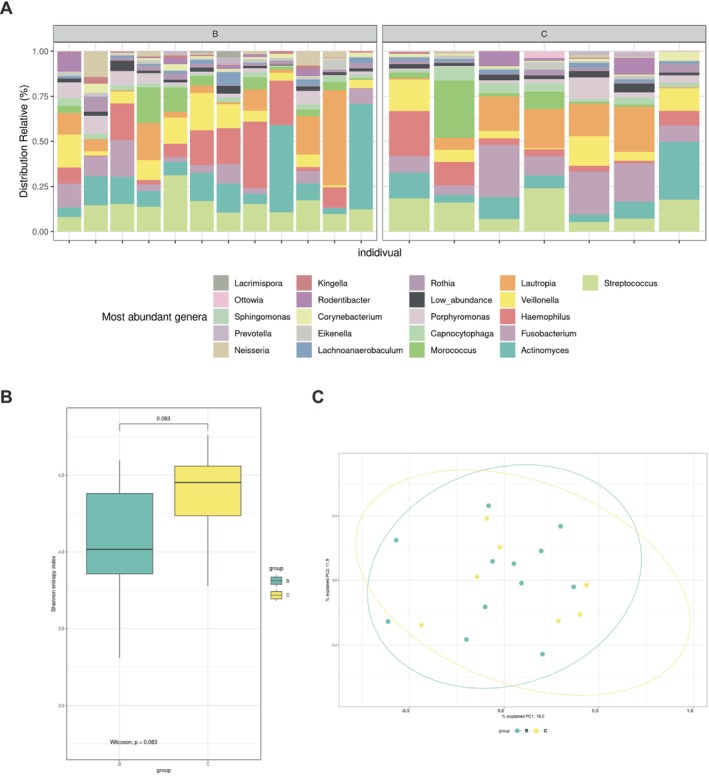
Participants with sleep bruxism have a limited reduction in supragingival microbiota α‐diversity. Supragingival microbiota samples from control participants and participants with sleep bruxism were harvested and subjected to 16S rRNA gene sequencing. (A) Relative abundance of must abundant genera. In panels B–C, amplicon sequence variants (ASV) analyses were performed to measure supragingival microbiota diversity and similarity. (B) ASV α‐Diversity (Shannon index) of each group. (C) Principal component analysis based on the Bray Curtis dissimilarity comparing the ASV from both groups. Samples from control participants are identified C while the samples from participants with sleep bruxism are identified B.

To study the association between supragingival microbiota composition and SB, bacterial DNA was extracted from supragingival microbiota samples collected from controls and individuals with SB. The samples were then subjected to 16S rRNA gene sequencing. Taxonomic annotation of the ASVs, at the genus level, revealed that the supragingival microbiota from SB individuals display different relative abundances than the controls (Figure 1a). Additionally, α‐diversity (Shannon index) and β‐diversity (Bray–Curtis dissimilarity index) were calculated based on the ASV abundance matrix. The Shannon index estimates the diversity of microbial species within a given sample, whereas the Bray–Curtis dissimilarity index measures the differences between samples from the two groups. We observed a strong trend (*p* = 0.083) toward less diverse microbiota (decreased α‐diversity) in the subgingival microbiota from individuals with SB compared to the controls (Figure 1b). Furthermore, the bacterial communities are clustering together, indicating no significant difference in β‐diversity between the groups (Figure 1c).

### 3.4 Supragingival Microbiota Alterations

**FIGURE 2:**
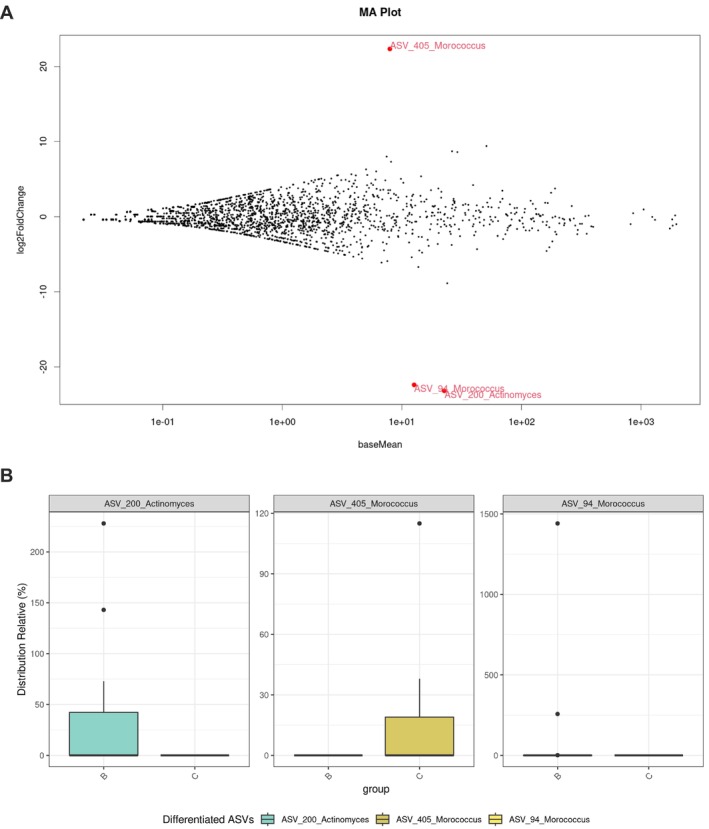
Participants with sleep bruxism present supragingival microbiota alterations. Amplicon sequence variants (ASV) differential analysis reveals a differential enrichment of ASV in the bruxism group. (A) MA Plot showing the distribution of ASV. Similar ASV are represented by black dots while the different ASV are represented by red dots. (B) Enrichment of ASV of microbiota from control participants and participant with sleep bruxism based on a differential enrichment analysis. Samples from control participants are identified C while the samples from participants with sleep bruxism are identified B.

To explore associations between supragingival microbiota composition and SB, ASVs were subjected to differential analyses. First, a principal component analysis (PCA) using the DESeq2 normalised data have been performed to visualise the variation between the ASVs. Figure S1 is showing that the ASVs from the control and bruxism groups are similar and clustering together indicating a good overall quality of the data. Second, a MA plot was used to visualise the results of the differential ASV analyses. We identified three ASVs that were differentially distributed between the groups (Figure 2a). Next, enrichment analyses revealed that ASV 405 (*Morococcus*) was more abundant in the control group, whereas ASVs 200 (*Actinomyces*) and 94 (*Morococcus*) were more abundant in the bruxism group (Figure 2b).

## 4 Discussion

Our study is, at the time of this writing, the first to explore the association between SB and supragingival microbiota composition. This approach opens new avenues of research into the underlying mechanisms of SB and its potential link to oral health. By identifying a correlation between supragingival microbiota composition and SB, our study will initiate future research aimed at understanding how alteration of the oral microbiota might influence the risk or severity of SB.

A recent study reported divergent β‐diversity in the whole saliva microbiota of patients with SB compared to control subjects [25]. The authors also observed a decreased relative abundance of *Streptococcus mitis* in the whole saliva samples collected from participants with self‐reported with clinical sign of probable SB. Although this study generated interesting results, caution should be applied in interpreting these data since the methodology lacks details on how the bacterial DNA was extracted and sequenced (number of base pair [pb], number of read pairs per samples, quality of the samples after sequencing, etc.) and on how the patients were selected in regard to sex and polygraphic measurements. In contrast to these findings, we observed a decrease bacterial diversity and an enrichment of ASVs related to *Actinomyces* and *Morococcus* genus in the supragingival microbiota samples from the SB group. The presence of distinct microbial ecosystems (or ecological niches) in the oral cavity explains these differences. Supragingival microbiota composition, a distinct ecological niche, differs greatly from that of the whole saliva microbiota, another distinct ecological niche, both in health and disease state [32].

Masticatory muscle activity during sleep has been associated with swallowing activity; and in SB individuals, 68% of swallowing activity occurred in conjunction with muscle activity, compared with only 10% in control subjects [33]. The rhythmic activity of the masticatory muscles is therefore often associated with a swallowing movement. We speculate that increased swallowing activity in SB participants may influence the composition of the supragingival microbiota. Additionally, SB is known to alter oral ecology, including the pH of the oral cavity and saliva production [34]. These changes may favour the growth of certain bacterial species at the expense of others, leading to reduced bacterial diversity. However, future studies are needed to confirm these hypotheses.

There is limited research characterising the genus *Morococcus* isolated from the oral cavity or other body sites. *Morococcus cerebrosus* has first been isolated from a brain abscess [35]. Several years later, Baris et al. [36] identified a bacterium from the genus *Morococcus* that contributed to the calcification of supragingival dental calculus in patients with periodontitis, and thereby influencing the composition of the supragingival microbiota via calculus. More recently, Urvashi et al. [37] isolated *M. cerebrosus* from the subgingival microbiota, another oral ecological niche, in patients with chronic periodontitis. Consistent with existing literature, we isolated the genus *Actinomyces* from the supragingival microbiota [32]. Implication of the *Actinomyces* genus in non‐oral disorders has also been described. For example, bacteria from the genus *Actinomyces* have been associated with neurodegenerative disorders such as Alzheimer disease [38]. Given that bacteria from the genera *Morococcus* and *Actinomyces* are understudied in relation to the mouth–brain axis and SB, further works are required to understand their contributions to this emerging field of research.

Inflammation may be the link between SB and subgingival microbiota composition. Systemic inflammation is known to modulate oral microbiota composition [20]. Systemic inflammation has also been associated with SB. In individuals with SB diagnosed by self‐report and clinical assessments, salivary level of cortisol was higher than in control subjects [39]. In urine samples of individuals with SB, concentration of 17‐hydroxycorticosteroids and C‐reactive protein correlated with bruxism episode index [40]. However, when the same research group investigated blood level of inflammatory markers in SB individuals, no differences were found compared to control subjects [41]. Metabolic syndrome, a condition characterised by chronic low‐grade inflammation, insulin resistance and cardiovascular risk factors, has been shown to influence both systemic and oral health, including microbiota composition [13, 42, 43]. Given its impact on inflammatory pathways, future research should explore whether metabolic syndrome exacerbates SB through alterations in immune response, muscle function, or oral microbial balance. Further works are also required to establish the link between SB, inflammation and subgingival microbiota composition.

Nevertheless, our study has several limitations. First, it is a unicentric study with a small number of participants per group. We followed the recruitment process from a similar study [29] and recruited 19 participants, which was 1 participant below our threshold of 20 participants. This small number of participants may impact data interpretation and data extrapolation at this time. Additionally, our findings focus on supragingival microbiota and SB in young, metabolically healthy subjects. Although this provides valuable insights into this specific population, it limits the generalizability of the results to other groups, such as those with underlying oral diseases (gingivitis, periodontitis, tooth decay, endodontic lesion, etc.) or systemic medical conditions. Finaly, the oral microbiota is known to be dynamic and subject to changes in response to various factors, such as diet, oral hygiene and medical treatments [11]. It is possible that some of these confounding factors were not fully controlled in our study, potentially affecting the internal validity of our results. Then caution is suggested before extrapolating our findings in relation to mechanism and causality of SB.

## 5 Conclusion

This study suggests that individuals with SB exhibit alterations in their supragingival microbiota compared to control subjects. By investigating the association between SB and supragingival microbiota, our research may lead to the assessment of important questions in the fields of oral microbiota composition and sleep medicine. For example, could microbiota help to dismantle the debate on the role of bruxism in periodontal disease [44, 45].

## Author Contributions

Athénaïs Collard: contributed to data acquisition and analysis, critically revised the manuscript; Alban Mathieu, contributed to data analysis, critically revised the manuscript; Paolo Landa: contributed to data analysis, critically revised the manuscript; Annik Pelletier: contributed to early conception of the study, critically revised the manuscript; Gilles J. Lavigne: contributed to conception and design, data acquisition, data analysis and interpretation, critically revised the manuscript; Arnaud Droit: contributed to data analysis, critically revised the manuscript; Nelly Huynh: contributed to conception and design, data analysis and interpretation, critically revised the manuscript; Vanessa P. Houde: contributed to conception, design, data analysis and interpretation, drafted and critically revised the manuscript. All authors gave final approval and agreed to be accountable for all aspects of the work.

## Conflicts of Interest

The authors declare no conflicts of interest.

## Supporting information


**Figure S1.** Principal component analysis from the DESeq2 normilized data comparing the ASV.

## Data Availability

Data from this study are available from the corresponding author on reasonable request.
