# Supplementary material for: Supragingival Microbiota Alterations in Individuals With Sleep Bruxism: A Pilot Study
Source: J Oral Rehabil. 2025 Apr 29;52(9):1319–27. doi: 10.1111/joor.13983 (PMC12408962; doi:10.1111/joor.13983)
Supplement: Supplementary file 1 — Figure S1. Principal component analysis from the DESeq2 normilized data comparing the ASV. [file JOOR-52-1319-s001.pdf]

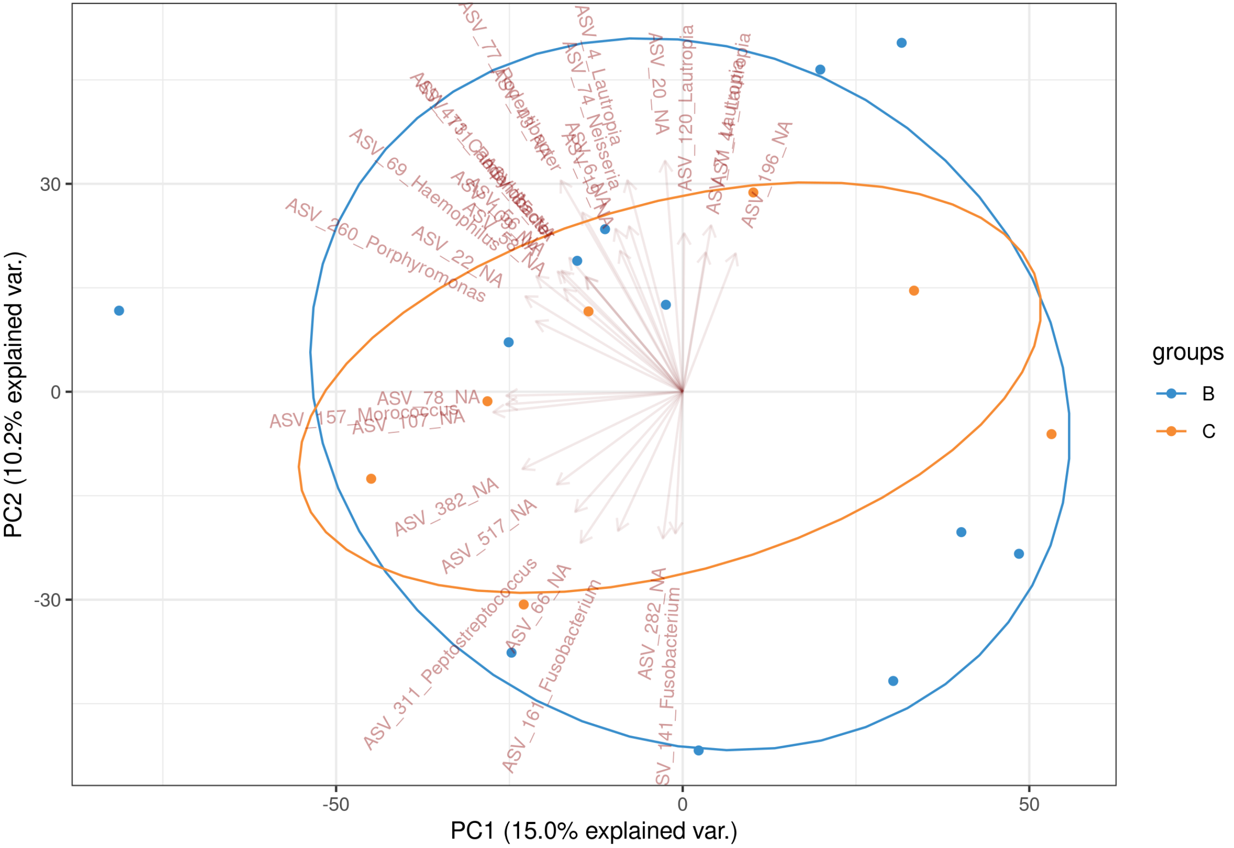

Supplementary Figure 1. Principal component analysis from the DESeq2 normilized data comparing the ASV from both groups. Samples from control participants are identified C while the samples from participants with sleep bruxism are identified B.
